# The Hypothermic Effect of Hydrogen Sulfide Is Mediated by the Transient Receptor Potential Ankyrin-1 Channel in Mice

**DOI:** 10.3390/ph14100992

**Published:** 2021-09-29

**Authors:** Emoke Olah, Zoltan Rumbus, Viktoria Kormos, Valeria Tekus, Eszter Pakai, Hannah V. Wilson, Kata Fekete, Margit Solymar, Leonardo Kelava, Patrik Keringer, Balazs Gaszner, Matthew Whiteman, Julie Keeble, Erika Pinter, Andras Garami

**Affiliations:** 1 Department of Thermophysiology, Institute for Translational Medicine, Medical School, University of Pecs, H-7622 Pecs, Hungary; potoneoe@gmail.com (E.O.); zoltan.rumbus@aok.pte.hu (Z.R.); eszter.pakai@aok.pte.hu (E.P.); kata.fekete@aok.pte.hu (K.F.); margit.solymar@aok.pte.hu (M.S.); leonardo.kelava@aok.pte.hu (L.K.); patrik.keringer@aok.pte.hu (P.K.); 2 Department of Pharmacology and Pharmacotherapy, Medical School, University of Pecs, H-7622 Pecs, Hungary; viktoria.kormos@aok.pte.hu (V.K.); valeria.tekus@aok.pte.hu (V.T.); erika.pinter@aok.pte.hu (E.P.); 3 Janos Szentagothai Research Centre, University of Pecs, H-7622 Pecs, Hungary; balazs.b.gaszner@aok.pte.hu; 4 Centre for Human & Applied Physiological Sciences, King’s College London, London WC2R 2LS, UK; drhannahwilson-nortey@outlook.com (H.V.W.); julie.keeble@kcl.ac.uk (J.K.); 5 Research Group for Mood Disorders, Department of Anatomy, Medical School and Center for Neuroscience, University of Pecs, H-7622 Pecs, Hungary; 6 Institute of Biomedical and Clinical Sciences, University of Exeter Medical School, University of Exeter, Exeter EX1 2LU, UK; m.whiteman@exeter.ac.uk

**Keywords:** hypothermia, thermoregulation, H_2_S, TRPA1, GYY4137

## Abstract

Hydrogen sulfide (H_2_S) has been shown in previous studies to cause hypothermia and hypometabolism in mice, and its thermoregulatory effects were subsequently investigated. However, the molecular target through which H_2_S triggers its effects on deep body temperature has remained unknown. We investigated the thermoregulatory response to fast-(Na_2_S) and slow-releasing (GYY4137) H_2_S donors in C57BL/6 mice, and then tested whether their effects depend on the transient receptor potential ankyrin-1 (TRPA1) channel in *Trpa1* knockout (*Trpa1^−/−^*) and wild-type (*Trpa1^+/+^*) mice. Intracerebroventricular administration of Na_2_S (0.5–1 mg/kg) caused hypothermia in C57BL/6 mice, which was mediated by cutaneous vasodilation and decreased thermogenesis. In contrast, intraperitoneal administration of Na_2_S (5 mg/kg) did not cause any thermoregulatory effect. Central administration of GYY4137 (3 mg/kg) also caused hypothermia and hypometabolism. The hypothermic response to both H_2_S donors was significantly (*p* < 0.001) attenuated in *Trpa1^−/−^* mice compared to their *Trpa1^+/+^* littermates. *Trpa1* mRNA transcripts could be detected with RNAscope in hypothalamic and other brain neurons within the autonomic thermoeffector pathways. In conclusion, slow- and fast-releasing H_2_S donors induce hypothermia through hypometabolism and cutaneous vasodilation in mice that is mediated by TRPA1 channels located in the brain, presumably in hypothalamic neurons within the autonomic thermoeffector pathways.

## 1. Introduction

Hydrogen sulfide (H_2_S) was considered to be an environmental toxin before it was identified as an endogenously produced mediator in the 1940s (for a review, see [1]). At present, H_2_S is recognized as an important gasotransmitter, which plays roles in a wide spectrum of physiological processes in the brain as well as in peripheral tissues in health and disease [2,3]. H_2_S is synthesized both centrally and peripherally by specific enzymes including cystathionine *β*-synthase, cystathionine *γ*-lyase, and 3-mercaptopyruvate sulfurtransferase, but alternative H_2_S-producing pathways are also known [3,4]. Endogenously, H_2_S is present within the nM to low µM concentration range and it is catabolized by enzymatic and non-enzymatic processes [5]. In the past decades, the role of H_2_S has been implicated in a number of physiological and pathological conditions [2,3,4], including the regulation of core body temperature (*T_b_*) [6].

In 2005, Blackstone et al. [7] reported that the inhalation of H_2_S induces concentration-dependent hypometabolism and hypothermia in mice without causing behavioral or functional damages to the animals. The authors hypothesized that the thermal effect was evoked through the H_2_S-induced inhibition of the terminal enzyme complex in the electron transport chain and called the condition as suspended animation-like state [7]. The hypometabolic and hypothermic response to H_2_S inhalation was also shown by other authors [8], and similar results were obtained by the administration of the H_2_S donor sodium hydrosulfide (NaHS) in anesthetized rats [9] and by infusion of dimethyl trisulfide in conscious mice [10]. However, the thermoregulatory role of H_2_S was debated later, when Hemelrijk et al. [11] could not reproduce the H_2_S-induced thermal effects and concluded that H_2_S exacerbates hypoxia-induced hypometabolism, but H_2_S in itself does not decrease metabolic rate and deep *T_b_*. The mediation of hypoxia-induced hypothermia by H_2_S was supported by the results of Kwiatkoski et al. [12], showing that production of H_2_S increases in the hypothalamus of rats exposed to hypoxia. Further complicating the issue, in larger animals the effects of H_2_S on metabolism and *T_b_* remained contradictory, because H_2_S-induced hypothermia was shown in pigs by some authors [13,14], whereas other groups did not find an effect on *T_b_* in pigs [15,16] and in sheep [17].

Several mechanisms underlying the thermoregulatory effects of H_2_S have been proposed (for review, see [6]), but the molecular site of action and the thermoeffector mechanism underlying H_2_S-induced hypothermia has remained unclarified. Peripheral and central inhibition of H_2_S-synthetizing enzymes were recently both shown to influence the fever response in rats, but neither of them altered deep *T_b_* in afebrile animals [18,19]. Within the central nervous system, endogeneous H_2_S production in the hypothalamus was shown to be involved in the hypothermia associated with endotoxic shock [20] and hypoxia [12]. However, injection of low doses of sodium sulfide (Na_2_S), i.e., an H_2_S-releasing salt, into the lateral ventricle or the medullary raphe did not cause any significant effect on *T_b_* of euthermic rats [19,21]. With regards to the thermoeffector mechanism, although it is well known that H_2_S plays a role in the regulation of the vascular tone [3,6], it is unknown whether skin vasodilation, a principal autonomic heat-dissipating thermoeffector [22], is recruited in the H_2_S-induced hypothermia. A plausible molecular target for a thermoregulatory effect could be a sensor that is expressed on neurons located within the thermoeffector pathways, therefore its stimulation can directly lead to a change in deep *T_b_* via modulation of the activity of one or more thermoeffector organs. Interestingly, H_2_S has been shown to interact with transient receptor potential (TRP) channels also including temperature-sensitive receptors, e.g., TRP ankyrin-1 (A1) and vanilloid-1 (V1) (for a review, see [23]), which channels are expressed within the neural thermoeffector pathways [22]. The TRPA1 channel can be of crucial importance for the thermal action of H_2_S, since TRPA1 channel-mediated effects of sulfide donors and polysulfide were identified in a plethora of experimental models used for the study of pain, inflammation, vasomotor responses, as well as neuronal, urinary, and cardiorespiratory functions.

An extensive list of studies that describeTRPA1-mediated effects of H_2_S was recently collected by Pozsgai et al. [24]. However, thermoregulatory effects were not mentioned by the authors. The diverse existence of H_2_S-induced TRPA1 activation in different homeostatic processes may suggest that it could also be involved in thermoregulation. In support of this possibility, TRPA1 was shown to be essential in the autonomic thermoregulatory response, particularly in cutaneous vasoconstriction, following cold exposure in mice [25]. Seemingly contradicting this finding is a previous study [26] that found that TRPA1 channels do not play a cold sensor role for autonomic thermoregulation in rodents. However, it is important to note that even if a TRP channel does not play a thermosensor role in the thermoregulation system, its modulation with ligands can still lead to changes of deep *T_b_*, as was shown in the case of another temperature-sensitive receptor, TRPV1 [27]. In summary, the question of whether and how the TRPA1 channel is involved in H_2_S-induced hypothermia has remained unanswered.

In the present study, we have investigated the thermoeffector mechanisms of the hypothermic response to fast- and slow-releasing H_2_S donors, then we used *Trpa1* knockout mice to show the contribution of TRPA1 channels to the response. We also studied TRPA1 expression in thermoregulation-related brain nuclei to explore the possible site of action for the hypothermic effect of H_2_S.

## 2. Results

### 2.1. Central Administration of Na_2_S Decreases Deep T_b_ in Mice via Inhibition of Thermogenesis and Induction of Vasodilation in the Skin

**Figure 1:**
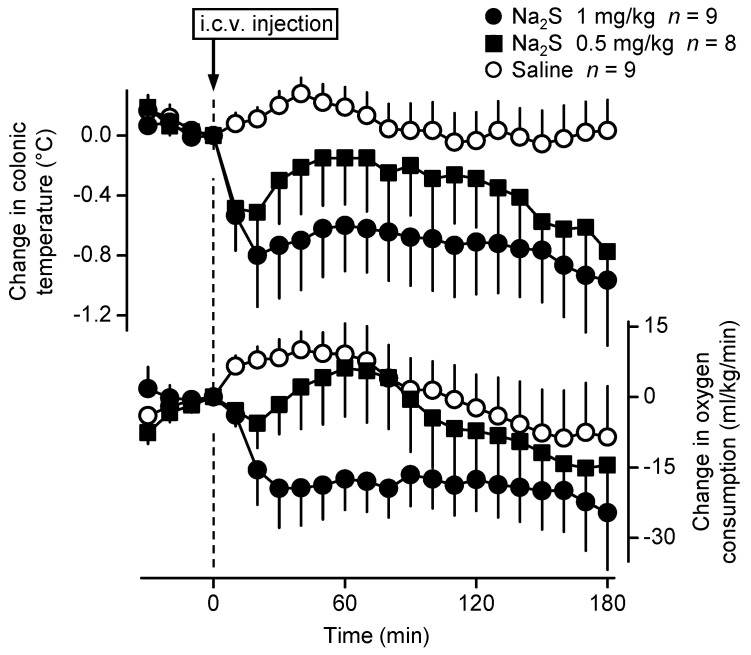
Colonic temperature and oxygen consumption (*V*O_2_) responses of C57BL/6 mice to Na_2_S (doses indicated) and saline administered i.c.v. The changes in colonic temperature (a form of deep *T_b_*) are shown in the upper panel, while the changes in *V*O_2_ (an indicator of thermogenesis) are depicted in the lower panel. Here and in Figures 2–5, *n* is the number of animals in each experimental group.

First, we studied the thermoregulatory effect of Na_2_S, a fast-releasing H_2_S donor [28], administered intracerebroventricularly (i.c.v.) in C57BL/6 mice by using respirometry thermometry (for details, see Experimental Setups in Materials and Methods). In response to Na_2_S, the mice developed a decrease in deep *T_b_*, which was more pronounced at the higher dose, whereas saline did not cause any effects (Figure 1).

The hypothermic response to Na_2_S developed rapidly at both of the applied doses, and at 20 min it reached the biggest mean decrease of −0.5 ± 0.3 °C at 0.5 mg/kg and −0.8 ± 0.3 °C at 1 mg/kg (*p* = 0.045 and 0.005, respectively). The effect of the treatment on *T_b_* was significant [ANOVA, *F*_(2506)_ = 41.158, *p* < 0.001] and so was the effect of time [ANOVA, *F*_(21,506)_ = 1.809, *p* = 0.015]. The effect was significant for both the lower and the higher doses of Na_2_S as compared to saline (*p* < 0.001 for both). At the 0.5 mg/kg dose of Na_2_S, deep *T_b_* was significantly lower than in saline-treated mice at 20, 170, and 180 min, while at the 1 mg/kg dose the drop in deep *T_b_* was significant between 20–180 min compared to saline.

In the same experiments, we also measured the rate of oxygen consumption (*V*O_2_), which was regarded as an indicator of non-shivering thermogenesis (i.e., one of the principal autonomic thermoeffectors), as in previous studies in mice [29,30]. The Na_2_S-induced hypothermia was brought about by a fall in *V*O_2_, which changed with similar dynamics as deep *T_b_* (Figure 1). Similar to *T_b_*, the effect of the treatment on *V*O_2_ was significant [ANOVA, *F*_(2506)_ = 28.860, *p* < 0.001] and so was the effect of time [ANOVA, *F*_(21,506)_ = 1.654, *p* = 0.034]. The effect was significant for both the lower and the higher doses of Na_2_S as compared to saline (*p* = 0.024 and *p* < 0.001, respectively). At the 1 mg/kg dose, *V*O_2_ was significantly lower than in saline-treated mice between 20 and 110 min. Since the expected effect was hypothermia, these experiments were performed at a subthermoneutral ambient temperature of 22 °C. Mice and rats exhibit cutaneous vasoconstriction in a subneutral environment as also indicated by their low tail-skin temperature [30,31], which did not allow us to study the potential contribution of skin vasodilation to the Na_2_S-induced hypothermia in this setup.

**Figure 2:**
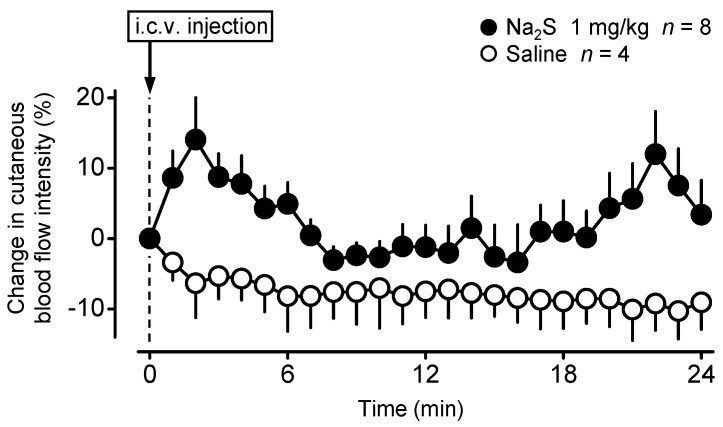
Blood flow intensity changes in the lumbar back-skin of anesthetized C57BL/6 mice in response to Na_2_S (dose indicated) and saline administered i.c.v.

In order to determine whether cutaneous blood perfusion is affected by central Na_2_S administration, as our next step, we measured changes of skin blood flow intensity on the lumbar back of anesthetized mice with laser speckle contrast imaging. The i.c.v. administration of Na_2_S (1 mg/kg) caused a prompt elevation in the cutaneous blood flow intensity, which reached the highest average of 14 ± 6% already at 2 min, then it decreased somewhat but remained higher than in the saline-treated mice throughout the experiment [ANOVA, *F*_(1250)_ = 74.081, *p* < 0.001] (Figure 2). The effect of time was not significant [ANOVA, *F*_(24,250)_ = 0.881, *p* = 0.629]. The blood flow intensity was significantly higher in response to Na_2_S compared to saline at 1–4, 6, and 20–24 min.

**Figure 3:**
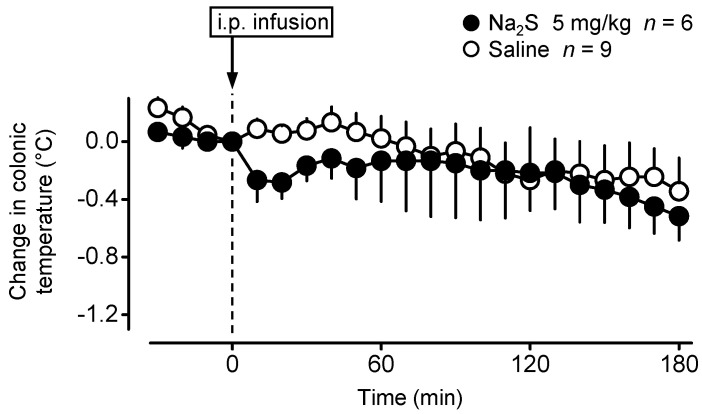
Colonic temperature response of C57BL/6 mice to Na_2_S (dose indicated) and saline administered i.p.

The rapid, dose-dependent development of the hypothermic and hypometabolic response to centrally administered Na_2_S suggests that the site of action for the released H_2_S is located in the central nervous system. To test whether the hypothermic effect of Na_2_S can be triggered from a peripheral site, next we studied the thermoregulatory response to the intraperitoneal (i.p.) administration of a high dose (5 mg/kg) of Na_2_S. As expected, i.p. infusion of saline did not have any effect on deep *T_b_* (Figure 3). In contrast with the i.c.v. administration, when the mice were infused with Na_2_S i.p. their deep *T_b_* did not differ significantly from that of saline-treated mice at any time points during the experiment (*p* > 0.05) even though a 10 times higher dose was delivered i.p. than the i.c.v. administered lower dose which caused hypothermia (see Figure 1). The effect of time was also not significant [ANOVA, *F*_(21,286)_ = 1.285, *p* = 0.183]

### 2.2. Central Administration of GYY4137 Decreases Deep T_b_ in Mice

**Figure 4:**
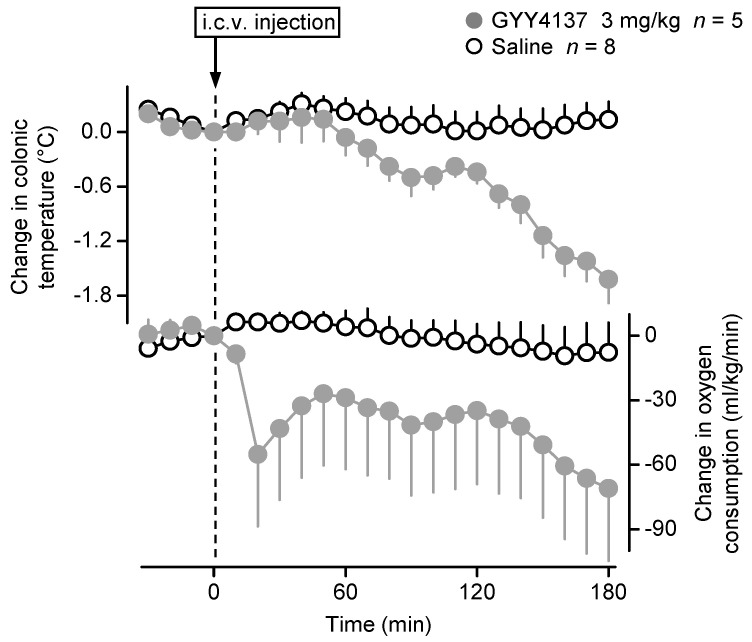
Colonic temperature and *V*O_2_ responses of C57BL/6 mice to GYY4137 (dose indicated) and saline administered i.c.v. Note that in the bottom graph the number of saline-treated mice is only 7, because in one of the animals the *V*O_2_ data could not be collected due to technical difficulties.

We also wanted to know whether the observed thermoregulatory effects of Na_2_S can be triggered by GYY4137, which is a slow-releasing H_2_S donor [28]. When administered i.c.v. in the respirometry thermometry setup, GYY4137 (3 mg/kg) caused a marked hypothermia and hypometabolism as compared to saline treatment (Figure 4). In accordance, the effect of treatment was statistically significant on both colonic temperature [ANOVA, *F*_(1242)_ = 108.220, *p* < 0.001] and *V*O_2_ [ANOVA, *F*_(1220)_ = 41.420, *p* < 0.001]. The effect of time was also significant on colonic temperature [ANOVA, *F*_(21,242)_ = 6.815, *p* < 0.001], but not on *V*O_2_ [ANOVA, *F*_(21,220)_ = 1.019, *p* = 0.441]. Between the GYY4137-treated and saline-treated mice, deep *T_b_* differed significantly at 80–100 min (*p* < 0.05) and 130–180 min (*p* ≤ 0.001), and the difference in *V*O_2_ was significant at 20–30 min and 160–180 min (*p* < 0.05).

### 2.3. The Hypothermic Response to Na_2_S and GYY4137 Is Attenuated in the Absence of the TRPA1 Channel

**Figure 5:**
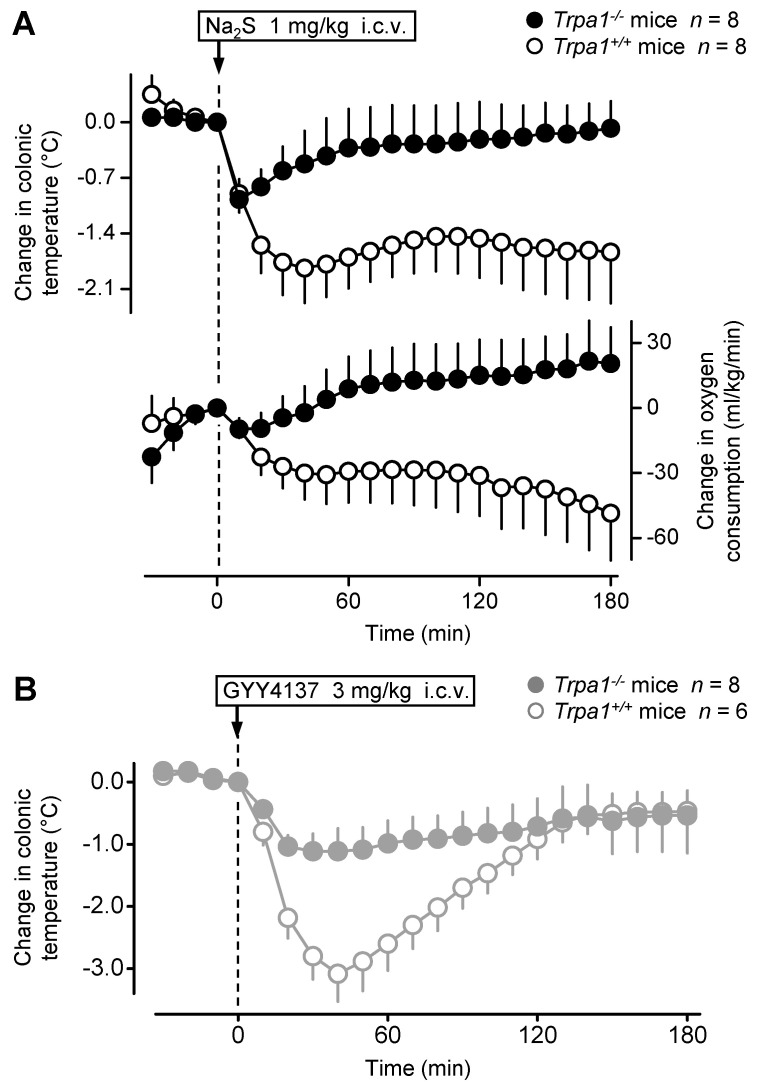
Colonic temperature (upper panel) and *V*O_2_ (lower panel) responses to Na_2_S (**A**) and colonic temperature responses to GYY4137 (**B**) administered i.c.v. (doses indicated) in *Trpa1^+/+^* and *Trpa1^−/−^* mice.

Previously it has been shown that the TRPA1 channel mediates different effects of H_2_S, including nociceptive, inflammatory, vasomotor, and neurophysiological effects (for a review, see [24]), but it has remained unknown whether it contributes to the development of the H_2_S-induced hypothermia. Therefore, next, we investigated whether the TRPA1 channel is involved in the thermoregulatory responses to different H_2_S donors. For that reason, we used mice with (*Trpa1^−/−^*) or without (*Trpa1^+/+^*) a homozygous mutation in the *Trpa1* gene and compared their hypothermic responses. As expected from our experiments in C57BL/6 mice (Figure 1), the i.c.v. administration of Na_2_S (1 mg/kg) caused a sudden, pronounced drop in the colonic temperature (>1.5 °C) and *V*O_2_ (>40 mL/kg/min) of the *Trpa1^+/+^* mice (Figure 5A). However, in the *Trpa1^−/−^* mice the hypothermic and hypometabolic effects of the same dose of Na_2_S were markedly attenuated [ANOVA, *F*_(1308)_ = 73.278, *p* < 0.001 and *F*_(1308)_ = 62.496, *p* < 0.001, respectively, for intergenotype difference]. The effect of time was also significant on colonic temperature [ANOVA, *F*_(21,308)_ = 2.535, *p* < 0.001], but not on *V*O_2_ [ANOVA, *F*_(21,308)_ = 0.173, *p* = 1.000]. The *Trpa1^+/+^* mice had significantly lower deep *T_b_* between 30 and 180 min, as well as reduced *V*O_2_ between 60 and 180 min post-Na_2_S administration as compared to the *Trpa1^−/−^* mice.

We also studied the thermoregulatory effect of GYY4137 in *Trpa1^−/−^* and *Trpa1^+/+^* mice in the thermocouple thermometry setup (Figure 5B). The i.c.v. administration of GYY4137 (3 mg/kg) caused a marked fall in the deep *T_b_* of *Trpa1^+/+^* mice; however, the hypothermic response to the same dose of GYY4137 was significantly attenuated in *Trpa1^−/−^* mice [ANOVA, *F*_(1264)_ = 31.819, *p* < 0.001]. The effect of time was also significant [ANOVA, *F*_(21,264)_ = 8.579, *p* < 0.001]. The colonic temperature of *Trpa1^−/−^* mice remained significantly higher than that of *Trpa1^+/+^* mice between 20 and 80 min post-GYY4137 administration.

### 2.4. Trpa1 mRNA Is Modestly Expressed in Brain Neurons within Autonomic Thermoeffector Pathways

Our thermophysiological results suggested that the thermoregulatory responses to H_2_S donors are triggered from the central nervous system. We, therefore, studied the expression of the TRPA1 channel in thermoregulation-related brain structures. Since the specificity of commercially available antibodies for the TRPA1 protein is debated [32,33], we measured its expression at the mRNA level with two different techniques: real-time-quantitative (RT-q)PCR and RNAscope in situ hybridization (for details, see Measurement of *Trpa1* mRNA Expression in Materials and Methods). Importantly, in both cases, the mice were carefully perfused before the brain sample collection. This step was required in order to avoid the contamination of the samples with blood, which was repeatedly shown to contain a detectable amount of TRPA1 channels [34,35,36]. First, we used RT-qPCR to assess *Trpa1* mRNA in the hypothalamus of the mice, but we did not find any detectable amount, although the expression of *Trpa1* mRNA was clearly present in the trigeminal ganglion, which was used as a positive control (Figure S1, Supplementary Materials).

**Figure 6:**
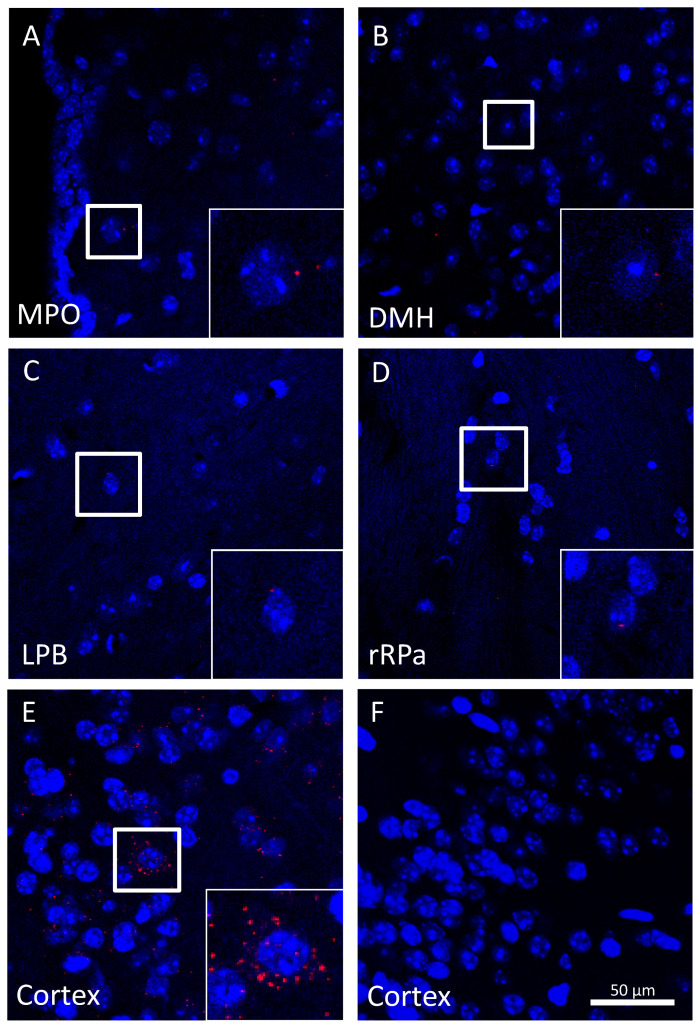
RNAscope in situ hybridization in the mouse brain for *Trpa1* mRNA. Representative confocal images of (**A**) the medial preoptic area (MPO), (**B**) the dorsomedial hypothalamic area (DMH), (**C**) the lateral parabrachial nucleus (LPB), (**D**) the rostral raphe pallidus nucleus (rRPa). Note the low number of *Trpa1* mRNA transcripts (red) in all areas as also shown in higher magnification insets representing the respective boxed areas (**A**–**D**). Positive control staining for *Ppib* mRNA (red) in the cortex (**E**) and negative control staining for the bacterial *dabP* mRNA (red) in the same cortical area (**F**). Cell nuclei were counterstained with DAPI (blue). Scale bar: 50 µm for all images.

Because the turnover of the TRPA1 protein in neurons may not necessitate a high rate of mRNA transcription, then we used RNAscope in situ hybridization, a highly sensitive method that can detect transcripts as single molecules [37]. We found detectable *Trpa1* mRNA transcripts in all of the studied thermoregulation-related nuclei, viz., in the medial preoptic area (Figure 6A), dorsomedial hypothalamic area (Figure 6B), lateral parabrachial nucleus (Figure 6C), and rostral raphe pallidus (Figure 6D), although it should be noted that the number of the *Trpa1* transcripts was low.

## 3. Discussion

In the present study, we show that fast- and slow-releasing H_2_S donors cause hypothermia which is mediated by reduced thermogenesis and increased cutaneous vasodilation. The hypothermic and hypometabolic effects are triggered from the central nervous system and both of them are strongly attenuated in the absence of the TRPA1 channel. TRPA1 channels located on hypothalamic neurons within autonomic thermoeffector pathways can be suggested as the molecular targets for the H_2_S-induced hypothermia.

First, we showed that injection of H_2_S donors into the lateral ventricle of the brain caused hypothermia and hypometabolism. These effects were more pronounced at higher doses in the case of Na_2_S. Our findings are in line with previous reports about the hypothermic response to H_2_S inhalation [7,8] and NaHS administration [9], whereas they contradict the results of other authors showing no significant thermoregulatory effect of centrally administered Na_2_S [19,21]. An explanation for the contradiction between the results obtained with Na_2_S can be that in the previous experiments Na_2_S was microinjected at low doses i.c.v. (870–1000 nmol/kg) [19] or into the medullary raphe (0.7–0.8 nmol/kg) [21], whereas in our study the doses were 6.4 and 12.8 µmol/kg and the lower dose of Na_2_S had modest thermoregulatory effects. Therefore, it is likely that Na_2_S was delivered at subthreshold concentrations to evoke a thermoregulatory effect in earlier studies. In accordance, NaHS also caused hypothermia when infused (to unspecified site) at a rate of 18–72 µmol/kg/h for 4 h [9]. It should be mentioned, however, that after the administration of the donors, H_2_S concentrations were not measured in the studies, thus a direct comparison of the H_2_S-induced effects at the different doses is not feasible. An alternative reason for the different findings can be that our experiments were performed in mice, whereas in the previous studies rats were used [19,21], which raises the possibility that the thermoregulatory effect of central H_2_S differs between mice and rats. Indeed, the lack of the hypothermic effect was reported in larger (non-rodent) animal species by different groups [15,16,17].

Next, we demonstrated that Na_2_S administered in the same way (1 mg/kg i.c.v.) as in the hypothermia experiments also increased the blood perfusion in the skin of the trunk (viz., lumbar back). Cutaneous vasodilation is an autonomic thermoregulatory response, which is recruited to decrease deep *T_b_* (or to prevent its elevation) through increased heat dissipation to the environment [22]. In small rodents, heat-exchange organs with non-hairy skin, e.g., tail and paws, are the predominant body parts for heat transfer between the body and the environment [38]. Our experiments were performed in anesthetized mice placed on a heating pad that was in contact with the ventral surface of the animal including its tail and limbs, therefore, the heating could have interfered with H_2_S-induced changes in blood flow in these body parts. On the contrary, the heating had minimal local effects on the vasculature in the skin of the lower back, thus we assessed the effects of H_2_S on the blood perfusion in that region. The use of the trunk blood perfusion for the assessment of heat dissipation is also justified by the recent finding that in mice the trunk contributes more to the whole-body heat loss than in rats and it is likely that the largest fraction of total heat loss comes from the body trunk in mice [39]. The involvement of skin vasodilation—in addition to reduced thermogenesis—in H_2_S-induced hypothermia indicates that H_2_S acts on two distinct efferent thermoeffector pathways or perhaps on neurons that are situated in the common afferent or efferent part of the warmth-sensitive pathways before it divides into separate branches to the different autonomic thermoeffectors. Theoretically, a direct effect of H_2_S on the vessels and the brown adipose tissue can be also mentioned, but that scenario is unlikely, since in our experiments the systemic (i.p.) administration of a high dose (10 times higher than what was effective i.c.v.) of Na_2_S did not have an effect on deep *T_b_* (Figure 3), thereby indicating a central (intrabrain) site for the thermoregulatory effects.

While Na_2_S is considered as a purer donor of H_2_S than NaHS, both substances yield H_2_S in a bolus instantaneously, which may question the physiological relevance of their effects (reviewed in [40]). In order to circumvent this issue, we also tested the thermoregulatory effects of GYY4137, a very slow-releasing H_2_S donor [41], which was used repeatedly to study the true physiological functions of H_2_S in a variety of experimental models, as reviewed by Whiteman et al. [42]. The i.c.v. administration of GYY4137 caused hypothermia and hypometabolism in the mice similar to Na_2_S. However, the dynamic of the response was different since the decrease in both *V*O_2_ and deep *T_b_* developed slower and its extent was more pronounced than in the case of Na_2_S. The difference in the dynamics of the hypothermia between GYY4137 and Na_2_S is well in harmony with their different capabilities of H_2_S generation, because H_2_S is released from GYY4137 in a slow and sustained manner, which was shown to evoke slow-onset vasodilatory effects [41].

Last, we wanted to discover the molecular target responsible for the hypothermic response to H_2_S. Temperature-sensitive members of the TRP channel superfamily can function as thermoreceptor elements in the thermoregulation system [22], but nonthermal activation of some of these TRP channels can also occur and contribute to the regulation of deep *T_b_* independently from whether the given channel is a thermosensor or not, as it was discovered in case of TRPV1 [43,44]. With regards to an interaction between H_2_S and thermosensitive TRP channels, strong evidence accumulated until present days for an action of H_2_S on the TRPA1 channel in a vast number of different experimental models [24], but whether TRPA1 also mediates the hypothermic effect of H_2_S was unknown until now. In the present work, we studied the thermoregulatory response to H_2_S donors (Na_2_S and GYY4137) in the genetic absence of the TRPA1 channel by using *Trpa1^−/−^* mice. We showed that the hypothermic and the hypometabolic responses are both attenuated in *Trpa1^−/−^* mice as compared to their *Trpa1^+/+^* littermates. The contribution of TRPA1 to the thermal effect of the H_2_S donors used in the present study is in harmony with our previous report about the hypothermic effects of a polysulfide, dimethyl trisulfide, which was also attenuated in *Trpa1^−/−^* mice [10]. However, polysulfides activate TRPA1 320 times more potently than H_2_S [45], thus it was crucial to understand whether H_2_S delivered by different non-polysulfide donors can evoke TRPA1-mediated hypothermia. The present findings clearly indicate, for the first time to our knowledge, that hypothermia induced by either fast- or slow-releasing H_2_S donors is mediated by the TRPA1 channel in unanesthetized mice.

Since in our experiments we also showed that the hypothermic response is triggered from the central nervous system, then we focused our attention on the expression of the TRPA1 channel in the brain. Because nonspecific binding was shown for different commercially available antibodies against the TRPA1 protein [33,46], we investigated the expression of the channel at the mRNA level in the hypothalamus of the mice, which brain region harbors high number of neurons within the autonomic thermoeffector pathways [47]. We did not detect the presence of *Trpa1* mRNA in the hypothalamus of mice with RT-qPCR. This finding is in accordance with a previous study, in which TRPA1 (formerly also called as ANKTM1) was not detected in the brain of mice [48]; however, it contradicts previous reports showing some TRPA1 expression in the hypothalamus of rats [49,50] and in the brain of mice [51,52]. It must be noted that in our experiments and in the study by Story et al. [48] the mice were perfused before the brain sample collection, whereas the animals were not perfused in any of the other studies [49,50,51,52], thus those results were also influenced by *Trpa1* mRNA originating from the components of the blood. In particular, TRPA1 was repeatedly detected in whole blood [34,35,36] and it is expressed in various peripheral blood leukocytes [53], monocytes [54], and lymphocytes [55,56]. In another study, extremely long exposure times for Northern blots were needed to detect *Trpa1* transcripts in the brain of humans suggesting low-abundance expression [57], but also questioning the sensitivity of the method for the detection of *Trpa1* mRNA. Considerable between-study differences in TRPA1 expression (3.6% vs. 56.5%) were also present in the dorsal root ganglia [48,58], which were presumed to be due to the detection sensitivity of in situ hybridization [59].

RNA integrity can be a critical issue in gene expression studies with qPCR, because fragmented RNA impairs qPCR amplification. The brain is characterized by a higher RNA degradation rate than other tissues [60], which also warrants for the need of more sensitive techniques to study gene expression in brain tissue. In contrast with qPCR, which requires the intact cDNA sequence (from the 5′ of the forward primer to the 3′ end of the reverse primer) for amplification, RNAscope requires the annealing of only 3 pairs of the 20 possible double Z probes to produce a detectable signal. Therefore, shorter mRNA molecules could still be detected by RNAscope probes in brain samples. In accordance with the higher sensitivity, with RNAscope we detected some *Trpa1* mRNA transcripts in all of the studied brain nuclei within the autonomic thermoregulatory pathways. The low-abundance expression of *Trpa1* mRNA in the studied thermoregulatory brain structures may indicate that these neurons play a minor role in the effect of H_2_S, but it has to be noted that mRNA expression does not necessarily correlate with the rate of protein translation, since a low mRNA expression can be associated with high protein levels, as shown in different studies [57,61]. Importantly, despite its low mRNA expression, TRPA1 is thought to play critical physiological functions in various tissues [57,61,62,63]. Similar to TRPA1, another temperature-sensitive TRP channel, TRPV3 was also shown to contribute to neurophysiological functions despite the low abundance of its mRNA in vagal afferent neurons [64]. It should be also mentioned that TRPA1 protein levels can be controlled via regulation of the protein’s lifetime by modulation of its ubiquitination status [57]. This may result in the presence of functional TRPA1 proteins on the neurons despite the low abundance of *Trpa1* mRNA. It was shown that de-ubiquitination of TRPA1 by the ubiquitin hydrolase protein CYLD increases the cellular pool of TRPA1 proteins [57] and CYLD expression was detected in different brain regions of mice, also including the hypothalamus [65]. Consequently, it is possible that the TRPA1 channel is expressed to a sufficient extent at the protein level to mediate the effects of H_2_S directly from the studied thermoregulation-related neurons. However, it cannot be excluded that TRPA1 channels in other brain structures are the primary sites for the action of H_2_S and modulate the activity of thermoeffectors through their projections to neurons within the thermoregulation pathways. In support of that scenario, physiological function for TRPA1 was found in the somatosensory cortex [66], in the hippocampus [63,66], as well as in the supraoptic and solitary nuclei [67,68], which brain regions are also involved in the regulation of deep *T_b_* [47,69,70].

The involvement of TRPA1 was shown in the development of hypothermia in response to diverse stimuli, such as acetaminophen [71], relative hypoxia [72], and thiazoline-related innate fear-eliciting compounds [73]. Although these agents should not be considered as selective activators of TRPA1, the hypothermic response to them was markedly attenuated or completely absent after the pharmacological or genetic blockade of TRPA1 channels yielding to the conclusion that activation of the TRPA1 channel by the applied stimuli mediates the hypothermia [71,72,73]. In one of these studies, the TRPA1 agonist cinnamaldehyde induced a marked hypothermia in *Trpa1^+/+^* mice, which was significantly reduced in *Trpa1^−/−^* mice [71]. These findings support our conclusions about the involvement of an H_2_S-induced activation of TRPA1 in the hypothermic response, but future research is warranted to reveal the exact nature of the interaction between H_2_S and TRPA1 and its contribution to the hypothermia.

Until the H_2_S-TRPA1 interaction in association with the hypothermic response is fully understood, alternative mechanisms of the hypothermic effect must be also stated. H_2_S can influence a variety of cellular structures and TRPA1 can be a channel that is important as a downstream mediator in different signal transduction pathways. In particular, the activation or expression of TRPA1 can be modulated by kinases, transcription factors, hormones, and reactive oxygen species, which can be activated by sulfides (for review, see [24]). Interestingly, through their activation in the central nervous system at least some of these signaling pathways can also contribute to a decrease in deep *T_b_* in response to different stimuli, as proposed, for example, for AMP-activated protein kinase [74], p38α mitogen-activated protein kinase [75], and estrogens [76]. Despite the involvement of TRPA1 in the signaling pathways, their activity does not necessarily depend solely on TRPA1, hence the blockade of the channel (e.g., in *Trpa1^−/−^* mice) may not lead to the inhibition of the whole pathway, which could explain why some (attenuated) hypothermic response to the used sulfide donors was still present in *Trpa1^−/−^* mice in our experiments. Whether H_2_S triggers hypothermia through the direct or indirect activation of TRPA1 channels remains the subject for future research.

## 4. Materials and Methods

### 4.1. Animals

Experiments were performed in 109 adult mice of both sexes. As in our earlier studies [10,26], male *Trpa1^−/−^* and *Trpa1^+/+^* mice (*n* = 18 and 14, respectively) were obtained from the Laboratory Animal Centre of the University of Pecs, where they were bred from breeding pairs generously donated by Dr. Pierangelo Geppetti. Seventy-seven C57BL/6 mice were also obtained from the Laboratory Animal Centre at the University of Pecs where they were bred and kept under standard pathogen-free conditions. The mice were housed in standard polycarbonate cages kept in a room with ambient temperature maintained at 24–25 °C and humidity at 30–40%. The room was on a 12 h light–dark cycle (lights on at 5:00 a.m.). Standard rodent chow and tap water were available ad libitum. At the time of the experiments, the *Trpa1^−/−^*, *Trpa1^+/+^*, and C57BL/6 mice weighed 26 ± 2, 26 ± 3, and 27 ± 4 g, respectively. For thermophysiological experiments, mice were extensively habituated to staying inside wire-mesh cylindrical confiners, as described previously [30]. All procedures were conducted under protocols approved by the Institutional Animal Use and Care Committee of the University of Pecs (registration no.: BA02/2000–6/2018, approved on 27 February 2018) and were in accordance with the directives of the National Ethical Council for Animal Research and those of the European Communities Council (86/609/EEC).

### 4.2. Surgeries

#### 4.2.1. Anesthesia and Perioperative Care

Mice were anesthetized with i.p. administration of a ketamine-xylazine cocktail (81.7 and 9.3 mg/kg, respectively) and received antibiotic protection intramuscularly (gentamycin, 6 mg/kg). During surgery, mice were heated with a temperature-controlled heating pad (model TMP-5a; Supertech Instruments UK Ltd., London, UK) placed under a surgery board. Each mouse was subjected to one of the surgical procedures described below. The experiments were performed 4–8 days after the surgery.

#### 4.2.2. I.c.v. Cannula Implantation

For the i.c.v. substance administration, A 22-G steel guide cannula (Plastics One, Roanoke, VA, USA) was implanted into the right lateral cerebral ventricle using a stereotaxic manipulator (Narishige Scientific Instruments Laboratory, Tokyo, Japan), as described previously [77]. In brief, after incision of the scalp and removal of the periosteum, two supporting microscrews (Fine Science Tools, Heidelberg, Germany) were driven into the skull and the guide cannula was inserted through a small hole drilled in the skull 0.5 mm posterior from Bregma and 1.0 mm lateral from midline. The tip of the cannula was placed within the right lateral ventricle (2.0 mm from dura). The cannula was fixed to the supporting microscrews with dental cement and closed by a dummy cannula.

#### 4.2.3. I.p. Catheter Implantation

For the i.p. administration of substances, a polyethylene (PE)-50 catheter filled with pyrogen-free saline was implanted into the peritoneal cavity, as described elsewhere [30,77]. Through a small midline incision on the abdominal wall, the internal end of the catheter was fixed to the abdominal wall with a suture, while the external end of the catheter was exteriorized at the nape and heat-sealed. The surgical wound was sutured in layers. The catheter was flushed with 0.1 mL of saline on the day after the surgery and every other day thereafter.

### 4.3. Experimental Setups

Thermoregulatory experiments in unanesthetized mice were performed in either the thermocouple thermometry setup or the respirometry thermometry setup. The experiments were conducted at an ambient temperature of 30 °C in the thermocouple thermometry setup and an ambient temperature of 22 °C in the respirometry thermometry setup, which is subneutral for mice in these setups [77]. Cutaneous blood flow measurement was performed by laser speckle contrast imaging in anesthetized animals.

#### 4.3.1. Thermocouple Thermometry

The mice were placed in cylindrical confiners and equipped with copper-constantan thermocouples (Omega Engineering, Stamford, CT, USA) to measure colonic temperature (a form of deep *T_b_*). The colonic thermocouple was inserted 3 cm deep beyond the anal sphincter, fixed to the base of the tail with adhesive tape, and plugged into a data logger device (Cole-Palmer, Vernon Hills, IL, USA) connected to a computer. Mice in their confiners were then placed into a temperature-controlled incubator (model MIDI F230S; PL Maschine Ltd., Tarnok, Hungary) set to an ambient temperature of 30 °C, which is slightly below the thermoneutral zone for mice in this setup. When the mouse was pre-implanted with an i.c.v. cannula, a needle injector (Plastics One, Roanoke, VA, USA) was fitted into the guide cannula and connected to a PE-50 extension, which was prefilled with a solution of Na_2_S or GYY4137 or with saline. The injector needle protruded 1.0 mm beyond the tip of the guide cannula. The extension was passed through a port of the incubator and connected to a 10-µL syringe (model 701N, Hamilton, Reno, NV, USA). When the mouse had an i.p. catheter, it was connected to a PE-50 extension, which was prefilled with the substance of interest and connected to a syringe placed in an infusion pump (model 975; Harvard Apparatus Inc., Holliston, MA, USA). The PE-50 extensions preloaded with the substances were wrapped in aluminum foil in order to prevent the photolytic oxidation of sulfide ions by UV light, which reaction can occur in aqueous sulfide solutions [78].

#### 4.3.2. Respirometry Thermometry

The mice were equipped with thermocouples and PE-50 extensions the same way as in the experiments in the thermocouple thermometry setup. Then, the mice in their confiners were transferred to a Plexiglas chamber of the four-chamber open-circuit calorimeter integrated system (Oxymax Equal Flow, Columbus Instruments, Columbus, OH, USA). The chambers were sealed, submerged into a temperature-controlled water bath, and continuously ventilated with room air (200 mL/min). The fractional concentration of oxygen was measured in the air entering and exiting the chamber, and the rate of oxygen consumption was calculated according to the manufacturer’s instructions using the Oxymax Windows software (version 3.1).

#### 4.3.3. Laser Speckle Contrast Imaging

The mice were anesthetized, then the fur on the lumbar back-skin was clipped and the animals were placed in a ventral position on a heating pad (model TMP-5a; Supertech Instruments UK Ltd., London, UK) to maintain their deep body temperature at 36 °C for the duration of the experiment. A needle injector was fitted into the preimplanted i.c.v. guide cannula and connected to a PE-50 extension, which was prefilled with a solution of Na_2_S or with saline. The blood flow intensity on the lumbar back-skin of the mice was measured by a PeriCam PSI system (Perimed AB, Järfälla, Sweden), which applies laser speckle contrast analysis technology. The system measures blood perfusion by recording changes in the speckle pattern as motion blurring in the regions of interests. If there is more movement in the region, e.g., due to higher red blood cell flow, blurring will increase and the speckle contrast will be lower, which correlates with blood flow. The change in blood perfusion (recorded in arbitrary perfusion units) during the experiments was expressed as percentage compared to the baseline value determined at the time of substance administration.

#### 4.3.4. Drugs and Drug Administration

Na_2_S nonahydrate (Na_2_S·9H_2_O) was purchased from Sigma-Aldrich (St. Louis, MO, USA). On the day of the experiment, Na_2_S·9H_2_O was freshly dissolved in pyrogen-free saline to achieve final concentrations of Na_2_S of 1.5, 5, or 10 mg/mL. For the i.p administration, the working solution (1.5 mg/mL) of Na_2_S (or saline) was infused over 4 min (3.3 mL/kg) to deliver Na_2_S at 5 mg/kg. For the i.c.v. administration of Na_2_S (at doses of 0.5 and 1 mg/kg), the working solutions (5 and 10 mg/mL, respectively) of Na_2_S (or saline) were infused (1 µL/min) over a 3 min period.

The slow-releasing hydrogen sulfide donor GYY4137 was synthesized at the University of Exeter Medical School, as described elsewhere [41]. On the day of the experiment, GYY4137 was freshly dissolved in saline to make a working solution of 30 mg/mL. By infusing this solution into the lateral ventricle (1 µL/min for 3 min), a total dose of 3 mg/kg of GYY4137 was delivered i.c.v. Control mice were infused with saline.

### 4.4. Measurement of Trpa1 mRNA Expression

#### 4.4.1. RT-qPCR

Three month-old male C57BL/6 mice (*n* = 7) and *Trpa1^−/−^* mice (*n* = 2, for negative control) were deeply anesthetized with an overdose of urethane (2.4 mg/kg) and perfused transcardially with 30 mL of phosphate-buffered saline (PBS). Brains and trigeminal ganglia were quickly dissected after decapitation, frozen on dry ice, and stored at −80 °C. Then, brains were sliced using razor blades on a coronal brain matrix (Ted Pella, Redding, CA, USA) to obtain 1 mm thick coronal sections, according to the technique described by Palkovits et al. [79]. A microdissecting tool (Ted Pella, USA) of 1 mm diameter was used to punch the hypothalamus between coronal planes of −0.5 mm to −2.5 mm caudal to Bregma, based on the atlas by Paxinos and Franklin [80]. Eight tissue punches were cut and pooled in order to collect samples representative of the entire hypothalamus of each animal. The microdissection procedure was performed on a dry ice-chilled mat and the punches were immediately snap-frozen in precooled Eppendorf vials on dry ice. Then, the samples were stored at −80 °C until the RNA isolation procedure.

Total RNA from the microdissected mouse brain samples and trigeminal ganglia were extracted with Direct-zol RNA Microprep kit (Zymo Research, Irvine, CA, USA). The concentration and purity of total RNA quality were assessed by a spectrophotometer (NanoDrop 2000, Thermo Fisher Scientific, Waltham, MA, USA). For further analysis, only those RNA samples were used that showed an A260/280 ratio between 1.9 and 2.1 and an A260/A230 ratio higher than 2.0. The RNA samples were treated with DNase I (Zymo Research, USA) to remove genomic DNA. Using Maxima First Strand cDNA Synthesis Kit (Thermo Fisher Scientific, USA), 1 μg RNA was reverse transcribed into cDNA. Applied Biosystems QuantStudio 3 RT PCR System (Thermo Fisher Scientific, USA) was used to perform qPCR experiments, using SensiFast SYBR Lo-ROX Kit (Bioline, Taunton, MA, USA) according to the manufacturer’s manual. All qPCR experiments were performed in technical replicates and included a melt curve analysis to ensure the specificity of the signal. Reverse transcriptase minus control showed the lack of genomic DNA contamination. The geometric mean of the reference gene Ct values was determined and *Trpa1* mRNA expression relative to the reference genes beta-actin (*Actb*) and glyceraldehyde-3-phosphate dehydrogenase (*Gapdh*) was calculated. Primers used to amplify target loci for RT-qPCR are listed in Table 1.

#### 4.4.2. RNAscope In Situ Hybridization

For RNAscope studies 3 month-old male C57BL/6 mice (*n* = 4) were perfused as described above using 30 mL PBS followed by 100 mL of 4% paraformaldehyde in Millonig’s phosphate buffer. Brains were postfixed for 24 h at room temperature, rinsed in PBS, dehydrated, and embedded in paraffin using standard procedures. 5 µm sections were cut using a sliding microtome (model HM 430; Thermo Fisher Scientific, USA). The RNAscope Multiplex Fluorescent Reagent Kit v2 (ACD, Hayward, CA, USA) was used according to the manufacturer’s protocol. In short, sections were heat-treated, deparaffinized, H_2_O_2_-blocked, boiled, and pretreated with Protease Plus. Subsequently, the sections were hybridized with probes specific to mouse *Trpa1* mRNA and with 3-plex positive and negative control probes. Sequential signal amplification and channel development were performed. Nuclear counterstaining with 4′, 6-diamidino-2-phenylindole (DAPI) was applied and sections were mounted with ProLong Diamond Antifade Mountant for confocal imaging. Probes and applied dilutions of fluorophores are listed in Table 2.

In accordance with earlier studies [81,82], cortical samples were stained for the *ppib* mRNA (red) as positive technical control (Figure 5E) and the bacterial *dabP* mRNA staining (red) was used as negative technical control (Figure 5F). According to the atlas by Paxinos and Franklin [80], fluorescent images of the medial preoptic area (+0.14 mm to +0.02 mm from Bregma), dorsomedial hypothalamic area (−1.58 mm to −1.70 mm from Bregma), as well as the lateral parabrachial nucleus and rostral raphe pallidus (−5.34 mm to −5.40 mm from Bregma for both) were acquired using an Olympus Fluoview FV-1000 laser scanning confocal microscope and Fluoview FV-1000S-IX81 image acquisition software system (Olympus, Tokyo, Japan). The confocal aperture was set to 80 µm. The analog sequential scanning was performed using a 40× objective lens (NA:0.75). The optical thickness was set to 1 μm and the resolution was 1024 × 1024 pixels. The excitation time was set to 4 µs per pixel. Blue and red virtual colors were selected to depict fluorescent signals of DAPI (nuclear counterstain) and of Cyanine 3 (*Trpa1* mRNA), respectively.

### 4.5. Data Processing and Analysis

Data on deep *T_b_*, *V*O_2_, and blood flow intensity were compared by two-way ANOVA. As in our previous studies [27,29], ANOVA was followed by the Fisher’s LSD post hoc test. Sigmaplot 11.0 (Systat Software, San Jose, CA, USA) software was used for statistical analyses. Differences were considered significant when *p* < 0.05. All data are reported as mean ±SE.

## 5. Conclusions

In conclusion, we show that slow- and fast-releasing H_2_S donors induce hypothermia through hypometabolism and cutaneous vasodilation in mice and that the hypothermic effect of H_2_S is mediated by TRPA1 channels located in the brain, presumably on hypothalamic neurons within the autonomic thermoeffector pathways. Our findings highlight the importance of central TRPA1-mediated H_2_S signaling in the thermoregulation system. In severe forms of systemic inflammation (e.g., septic shock), which is often associated with hypothermia [83] and by enhanced production of H_2_S [40,84], the interaction between TRPA1 and H_2_S can play a crucial role in the development of the response and, as perspective, may serve as a therapeutic target. Furthermore, the H_2_S-induced activation of central TRPA1 channels may pave the road to the development of controlled induction of hypothermia, but future research is needed to reveal the true thermopharmacological potential of the central TRPA1-H_2_S interaction in health and disease.

## Figures and Tables

**Table 1:** Primers used to amplify target loci for RT-qPCR.

Gene Amplified (Mus Musculus)	Nucleotide Sequence of Primer	Primer Type	Product Length in bp	NCBI RefSeq
*Trpa1*	ATCCAAATAGACCCAGGCACG	sense	101	NM_177781.5
	CAAGCATGTGTCAATGTTTGGTACT	antisense		
*Gapdh*	TTCACCACCATGGAGAAGGC	sense	237	NM_001289726.1
	GGCATGGACTGTGGTCATGA	antisense		
*Actb*	CTGTATGCCTCTGGTCGTAC	sense	214	NM_007393.5
	TGATGTCACGCACGATTTCC	antisense		

**Table 2:** RNAscope probes and applied dilutions.

Probes (Mus Musculus)	Catalog Number	Fluorophores	Dilution
*Trpa1*	400211	TSA Plus Cyanine 3	1:750
3-plex Negative Control Probe	320871	TSA Plus Fluorescein, Cyanine 3 and 5	1:750
3-plex Positive Control Probe	320881	TSA Plus Fluorescein, Cyanine 3 and 5	1:750

## Data Availability

Data is contained within the article and Supplementary Materials.

## Supplementary Materials

The following are available online at https://www.mdpi.com/article/10.3390/ph14100992/s1, Figure S1: Representative electrophoretogram of the RT-qPCR products.
