# Supplementary material for: The Hypothermic Effect of Hydrogen Sulfide Is Mediated by the Transient Receptor Potential Ankyrin-1 Channel in Mice
Source: Pharmaceuticals (Basel). 2021 Sep 29;14(10):992. doi: 10.3390/ph14100992 (PMC8538668; doi:10.3390/ph14100992)
Supplement: Supplementary file 1 [file pharmaceuticals-14-00992-s001.zip › pharmaceuticals-1374787-supplementary.pdf]

Supplementary Material

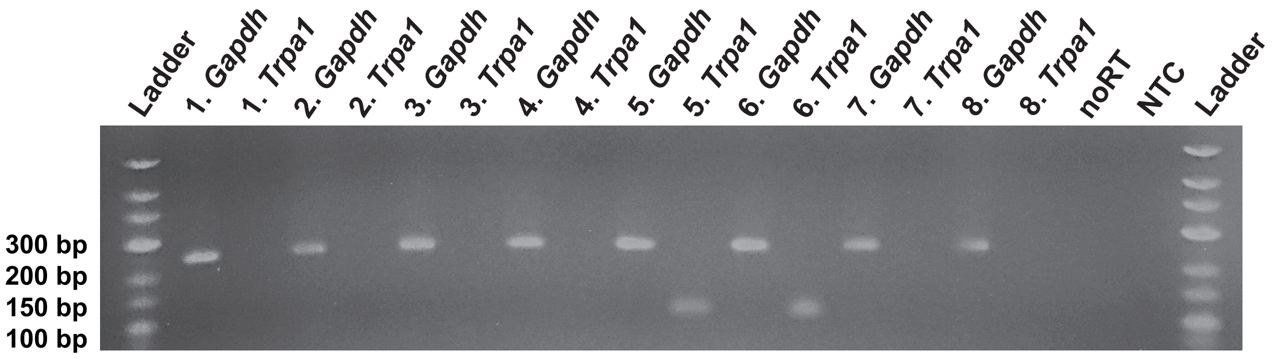

Figure S1. Representative electrophoretogram of the RT-qPCR products. Samples 1-4 represent the expression of the studied genes (*Gapdh* and *Trpa1*) in the hypothalamus of C57BL/6 mice. Samples 5 and 6 represent the expression of the genes in the trigeminal ganglia of C57BL/6 mice (positive controls), while samples 7-8 represent the gene expressions in the trigeminal ganglia of *Trpa1*$^{-/-}$ mice (negative controls). Note that the housekeeping gene *Gapdh* (size: 237 bp) was expressed in all samples, whereas the gene of interest *Trpa1* (size: 101 bp) was detectable only in the trigeminal ganglia of C57BL/6 mice. No reverse transcriptase (noRT) and no template controls (NTC) are shown as technical controls.
